# Non-prescribed sale of antibiotics for acute childhood diarrhea and upper respiratory tract infection in community pharmacies: a 2 phase mixed-methods study

**DOI:** 10.1186/s13756-018-0389-y

**Published:** 2018-07-31

**Authors:** Daniel Asfaw Erku, Sisay Yifru Aberra

**Affiliations:** 1 School of Pharmacy, University of Gondar, Lideta kebele 16, P.O.Box: 196, Gondar, Ethiopia; 2 College of Medicine and Health Sciences, University of Gondar, Gondar, Ethiopia

**Keywords:** Community pharmacy, Simulated patient, Antimicrobial, Dispensing Ethiopia

## Abstract

**Background:**

Although prohibited by law and legal regulatory frameworks, non-prescribed sale of antibiotics in community medicine retail outlets (CMROs) remains a serious problem in Ethiopia. The aim of this study was to document the extent of and motivations behind non-prescribed sale of antibiotics among CMROs in Gondar town, Ethiopia.

**Methods:**

A 2 phase mixed-methods study (a simulated patient visit followed by an in-depth interview) was conducted among CMROs in Gondar town, Ethiopia. Two clinical case scenarios (acute childhood diarrhea and upper respiratory tract infection) were presented and the practice of non-prescribed sale were measured and results were reported as percentages. Pharmacy staff (pharmacists and pharmacy assistants) were interviewed to examine factors/motivations behind dispensing antibiotics without a valid prescription.

**Results:**

Out of 100 simulated visits (50 each scenarios) presented to drug retail outlets, 86 cases (86%) were provided with one or more medications. Of these, 18 (20.9%) asked about past medical and medication history and only 7 (8.1%) enquired about the patient’s history of drug allergy. The most frequently dispensed medication for acute childhood diarrhoea simulation were oral rehydration fluid (ORS) with zinc (*n* = 16) and Metronidazole (*n* = 15). Among the dispensed antibiotics for upper respiratory infection simulation, the most common was Amoxicillin (*n* = 23) followed by Amoxicillin-clavulanic acid capsule (*n* = 19) and Azithromycin (*n* = 15). Perceived financial benefit, high expectation and/or demand of customers and competition among pharmacies were cited as the main drivers behind selling antibiotics without a prescription.

**Conclusions:**

A stringent law and policy enforcement regarding the sale of antibiotics without a valid prescription should be in place. This will ultimately help to shift the current pharmacy practices from commercial and business-based interests/practices to the provision of primary healthcare services to the community.

**Electronic supplementary material:**

The online version of this article (10.1186/s13756-018-0389-y) contains supplementary material, which is available to authorized users.

## Background

The development of antimicrobial agents represents one of the most significant achievements of modern medicine in the past century. Yet, the emergence of antimicrobial resistance (AMR) combined with the downturn in the introduction of new antibiotics to the market poses an unanticipated threat in the treatment of infectious diseases [1]. The emergence and spread of antimicrobial resistance is largely attributed to the use, over use or misuse of antimicrobials [2]. Community medicine retail outlets (CMROs) represent one of the main sources of antimicrobials globally [3]. According to the recent multi-country public awareness survey conducted by the World Health Organization (WHO), 93% of people got their most recently taken antimicrobial from a pharmacy and drug store [4]. In Ethiopia, the widespread access and availability of antibiotics without a valid prescription, coupled with the general public’s poor knowledge about antimicrobials, has increased self-medication with these drugs and augmented the risk of the inappropriate antibiotics use in the community [5–7]. Non-prescribed sale and dispensing of antibiotics will not only promote antimicrobial resistance but can also be linked with adverse drug events, significantly increased cost burden and poor health outcome. Thus, by acting as antimicrobial stewardship proponents, community pharmacists can play a significant role in the containment of cost and antimicrobial resistance.

Antimicrobial stewardship (AMS) is the term collectively used for a variety of quality improvement activities aimed at improving and sustaining the appropriate use of antibiotics for the treatment and/or prevention of infectious diseases [8]. One approach to achieving this is through the development of formal institutional AMS programs. The regular correspondence that community pharmacy professionals have with patients, their responsibility for dispensing these agents as well as the training and knowledge they possess, provides a great deal of opportunity to implement interventions for patients in order to slow antimicrobial resistance, verify appropriate usage, recommend alternative over-the-counter (OTC) medications, and educate patients. The majority of AMS programs in Ethiopia have been introduced in institutional settings such as hospitals and health management organizations. In contrast, there are no established antibiotic stewardship programs that formally exist in CMROs. Moreover, a recent national survey conducted in Ethiopian community pharmacies identified a need for incorporation of AMS program in CMROs and implement educational intervention to improve over the counter sale of antimicrobials and reduce the occurrence of antimicrobial resistance in the community [9].

This study is part of a larger investigation focusing on implementing a community pharmacy-based antimicrobial stewardship program in Ethiopia. In this mixed-methods study, we employed a simulated client method to document the extent of non-prescribed sale of antibiotics for the two most common minor ailments encountered in pharmacies (acute childhood diarrhea and uncomplicated upper respiratory infection) [10]*.* We then employed a qualitative study, using the theory of planned behaviour, to examine factors/motivations behind dispensing antibiotics without a valid prescription. The overarching aim of this study was, therefore, to document the extent and drivers of non-prescribed sale of antibiotics among CMROs in Gondar town, Ethiopia.

## Methods

### Study design and setting

A 2 phase mixed-methods research design, including a simulated client method (August 2017) and an in-depth interview with community pharmacists (October 2017) was used to comprehensively assess pharmacists’ overall practice regarding over the counter sale (OTC) of antibiotics in community pharmacies. The study was conducted in Gondar town, Northwest Ethiopia. The town has a population of approximately 206,987 [11] and the town has 57 CMROs (20 community pharmacies, 35 drug stores and 2 rural drug vendors). Ethical approval to conduct this study was granted by the ethical review committee of School of Pharmacy, University of Gondar. Informed consent was obtained from all participants for the qualitative study. The obtained participant’s information were kept confidential.

### The simulated patient study

Drug retail outlets in Ethiopia are classified based on the type of medications they are supposed to stock/dispense and qualification of pharmacy staff. Pharmacies were defined as drug retail outlets having at least one licensed pharmacist with a minimum qualification of a bachelor of pharmacy as a pharmacy staff, whereas drug stores are drug retail outlets having at least one licensed pharmacy technician/druggist with a minimum qualification of a diploma in pharmacy as a pharmacy staff. All pharmacies and drug stores located in Gondar town (a total of 50 CMROs) were visited. As each CMROs were visited twice by each simulated patient (SP), a total of 100 simulated visits were conducted over a 2-weeks period.

Two different clinical case scenarios, which are considered to be common (acute childhood diarrhea and uncomplicated upper respiratory infection) were developed and enacted by the simulated patients. In the acute diarrhea scenario, a female simulated patient (31-year-old) asked the pharmacy staff to give her a medication to relieve the acute diarrhea suffered by her 3-year-old son. The scenario was designed to exclude acute bloody, persistent diarrhea and diarrhea due to malnourishment. In the uncomplicated upper respiratory tract infection (URTI) scenario, a male simulated patient (27-year-old) asked for a medication after presenting with a symptom of upper respiratory tract infection. Details of the scenarios, along with the data collection tool used by the SPs are presented in Additional file 1. In both scenarios, the SP noted the queries and recommendations provided by the pharmacist including drug allergies, non-pharmacological advices given and medications dispensed.

Four clinical pharmacists acted as simulated patients. A half-day long discussion and training was given to SPs so that they will be familiar and be able to perform the clinical scenarios given. They were instructed not to give and/or ask further information unless asked by the pharmacy staff so as to make sure that the information provided by each SPs is uniform across all the visits. In order to avoid depending on the human cognitive processes, which has been mentioned as a potential limitation of the simulated patient method [12], all the visits were audio recorded. Immediately after each visit, the SPs filled the data gathered in a form containing a check list of items (such as queries/patient history requested, medications and/or counselling delivered and duration of the visit) that were intended to assess the practice of pharmacy personnel toward the dispensing of antibiotics for the specified minor ailments. The principal investigator (DAE) compared and validated the data from the check list against audio recordings for the purpose of quality assurance. Data were entered into and analyzed using Statistical Package for Social Studies (SPSS) version 20 for Windows and results are presented as frequencies and percentages.

### The qualitative study

In the second phase of the study, we employed a qualitative study using in-depth interviews to examine factors/motivations behind dispensing antibiotics without a valid prescription The interviews were constructed based on the theory of planned behaviour (TPB). TPB is a theory in psychology that links people’s belief and their behaviour. It assumes that individuals’ attitudes, subjective norms, perceived behavioural control and moral obligation ultimately determines their behavioural intention and/or how they display the behavior [13]*.* TPB has been employed extensively in pharmacy practice research [14, 15]. To allow for a maximum variation and come up with a richer data [16]*,* we recruited pharmacy staff from different types of drug retail outlets (considering the socioeconomic status of customers served in these CDROs), geographical location (urban/rural), and varied demographics (such as age, gender, educational level and experience in community pharmacy). From a total of 17 pharmacy staff invited, 15 pharmacy staff (9 pharmacists and 6 pharmacy assistants) agreed to participate. Based on the preliminary data analysis, the data was saturated after 13th participant was interviewed, after which no new content was identified [17]*.* All interviews were conducted by the principal author (DAE) in person at either the participant’s work place or other convenient locations such as coffee shops. Interviews were recorded and transcribed verbatim immediately following after each interview.

Each interview took approximately 30–45 min. Thematic analysis were performed using individual narratives of interviewed pharmacy staff as the main unit/case of analysis. The content of the interviews were carefully identified and coded in light of the TPB theory constructs, which then yielded the main themes of the analysis. NVivo 11 Software was used to assist the coding process.

## Results

### Finding of simulated visits

Out of 100 simulated visits (50 scenarios each) presented to drug retail outlets, 86 cases (86%) were provided with one or more antimicrobials. Of the 86 drug retail outlets selling an antibiotic when childhood diarrhea and upper respiratory tract infection was simulated, only 7 (8.1%) enquired about the patient’s history of drug allergy, 18 (20.9%) asked about past medical and medication history. Only 9 (10.6%) of the community pharmacy professionals advice simulated patients to visit a physician and only 12 (14%) of them provided advise on non-pharmacological management. The average time taken for pharmacy staff to counsel and dispense were 4 min. The detailed actions and advises provided by the pharmacy staff is presented in Table 1.

**Table 1:** Actions and advice in response to SPs in Gondar, Northwest Ethiopia, 2017

Type of action and advice	Total	Childhood diarrhea (*n* = 50)		URTI (*n* = 50)	
		Pharmacy (*n* = 28)	Drug store (*n* = 22)	Pharmacy (*n* = 28)	Drug store (*n* = 22)
Dispensed antimicrobial (s) without prescription	86	21	19	22	24
Asks drug allergies	7	3	–	4	–
Instruction on dose and duration	36	12	7	11	6
Instruction on side effects	24	13		8	3
Queries about past medical and medication history	18	7	1	10	–
Need for prescription	7	4		3	
Advice to visit physician	9	3	2	3	1
Non-pharmacological advice	12	4	2	3	3

Abbreviation: URTIs: Upper Respiratory Tract Infections

A variety of medications were dispensed for acute diarrhea including antiamoebic, antibiotics, anthelmintic, oral rehydration salts (ORS) and zinc (Table 2). Similarly, a wide variety of antimicrobial agents were dispensed and/or recommended in simulated scenarios. Among the dispensed antibiotics, the most common was Amoxicillin [18] followed by Amoxicillin-clavulanic acid capsule [19], Metronidazole [15] and Azithromycin [15].

**Table 2:** Medications dispensed in response to the simulated scenarios, Gondar town, Ethiopia, 2017

Antimicrobial (s) dispensed	Acute diarrhea	URTI
Cotrimoxazole	11	–
Metronidazole	15	–
Mebendazole	10	–
Loperamide	9	–
ORS with Zinc	16	–
Amoxicillin	–	23
Amoxicillin-clavulanic acid capsule	–	19
Azithromycin	–	15
Ciprofloxacin	–	5
Cephalexin	–	1
Cefexime	–	1
Levofloxacin	–	3

Abbreviation: ORT: Oral Rehydration Therapy; URTI: Upper Respiratory Tract Infections

### Motivation behind non-prescribed sale of antibiotics

Majority of pharmacy staff who were interviewed reaffirmed the finding from the simulated patient visits that non-prescribed sale of antibiotics is a common practice. This malpractice seems to be driven by a variety of factors which are summarized as behavioural belief (perceived financial benefit) and normative beliefs (high expectation of customers and competition among pharmacies).

### Perceived financial benefit

The lucrative financial benefit gained from the sale of antimicrobial agents whenever the patient requested them is discussed across all participants. There were also instances where participants indicated that their employers (pharmacy owners) expect them to do what is most profitable to the company including dispensing antibiotics without a valid prescription.

*“I am particularly concerned about the financial consequences for my pharmacy. It becomes difficult these days to be a pharmacy owner and gain some financial benefits since everybody even the nurses are taking away our business. So I do whatever it takes to cope up with such financial pressure and this includes handing over antibiotics and other medications without a doctor’s prescription (P1)”.*

*“Yes, it is very unprofessional to dispense these drugs (antibiotics) irrationally, but the pressure is very immense…you have an employer here that tells you constantly to dispense without a prescription. I don’t want to rescue my job by not doing so. Besides, a nearby pharmacy is voluntary to dispense even if I said no. Thus I usually sale this products to our customers (P7)”.*

### High expectation of customers and competition among pharmacies

Perceived beliefs regarding customers’ expectation and beliefs about the practice of other pharmacies were the two main normative beliefs held by the majority of the participants. One participant pointed out that their pharmacy business model is in such a way that customers satisfaction is the central core value.

*“We believe in customer satisfaction, this is something we developed over years of experience. If we insist not selling these products to them, we definitely loss these customers because they will be dissatisfied. Thus, we are usually pretty much encouraging for my employees to give them what they asked with the correct instruction (P8)”.*

*“Everyone comes with a request of either Amoxicillin or Amox/Clav and it looks like they already used it once or twice before and are very much confident about what they are asking for. It’s just impossible to say no when over half of your profit comes from selling antibiotics. I would say it is a matter of constant customer demand and financial consequence (P2)”.*

The perceived belief that customers can easily access antibiotics from neighbouring drug retail outlets appears to be one of the main drivers behind the pharmacists’ dispending behaviour.

*“It is very simple. If I am not happy to sell them (customers) these products, they will go to the neighboring pharmacy and easily get them easily without a question. So it becomes a matter of competition and retaining customers (P11)”.*

## Discussion

In most of the developing countries, the sale of antimicrobials in community medicine retail outlets is largely unregulated, without involvement of a licensed pharmacy personnel, and is often without a valid medical prescription [3]. This is the first comprehensive sequential mixed-methods study to document the extent and drivers of non-prescribed sale of antimicrobials in community pharmacies in Ethiopia. We employed a simulated patient methodology in phase 1, which is a more reliable approach if pharmacy staffs could be tempted to hide their actual behavior and practice due to a potential desirability bias [20]. Our use of a simulated patient method allowed us to evaluate the actual practice of a pharmacy staff when confronted with the choice of dispensing antimicrobials without a valid prescription, rather than assessing their performance with self-reported surveys, which is subjected to a number of potential biases. We also employed an in-depth interview with the aim of uncovering the drivers/motivations behind OTC sale of antibiotics. Although illegal and prohibited by legal regulatory frameworks, dispensing antibiotics without a valid prescription remains a serious problem in Ethiopia, as in many other African countries such as Zambia, Nigeria and Tanzania [19–21]. During the simulated visits, antibiotics were obtained without a valid prescription with ease from more than two third of pharmacies and drug stores of Gondar town. Furthermore, antibiotics were handed over to simulated patients with negligible queries regarding details of symptoms, past medical and medication history, possible drug allergies and the potential side effects of the dispensed medications. Similar studies conducted in many low and middle income countries (LMICs) also reported comparable findings [19–22]. In Tanzania, antimicrobial agents were dispensed without a prescription to 81% of simulated diarrhea cases and 95% of simulated upper respiratory infection [19]. Similarly, studies conducted in Zambia and Uganda reported that non-prescribed sale of antibiotics is widespread and frequent [20, 22]. Studies conducted in a number of other countries including Greece and Spain also showed that dispensing of antibiotics without a prescription is a common practice [18, 23].

In Ethiopia, as in many other countries [21, 24–26], antimicrobial agents are classified as prescription-only medicines and they can only be accessed, procured, and/or dispensed with a valid medical prescription. A number of factors could be cited for the higher rate of non-prescribed sale of antibiotics in the study area. One reason could be to the fact that most pharmacy staffs are more engaged with making sales and serving their commercial interests than providing primary healthcare services to the public. This notion is further supported by the study conducted in Saudi Arabia, where pharmacy staffs dispensed antimicrobial agents without even being requested [24]. Similar other studies conducted in Ethiopia and other African countries cited continued client demand as a major reason for perpetuating non-prescribed sales of antibiotics [21, 27, 28]. Another potential reason for widespread antibiotic dispensing in CMROs could be the market financial incentives where antimicrobial agents may be more profitable than evidence based, better and rational management options for minor ailments such as acute diarrhea and upper respiratory infections [29].

The potential benefits gained from investing in multifaceted interventions to protect the general public from the consequences of antibiotic misuse/overuse (such as antimicrobial resistance and increased healthcare cost) can outweigh the potential perils. Although a comprehensive pharmacy-based educational intervention is essential to curb inappropriate use of antibiotics [30], it should only be a part of a more comprehensive, multifaceted strategies and measures, which may include increasing awareness of rational use of antibiotics and antimicrobial resistance among the community, strictly implementing national regulations governing OTC sale of antibiotics and promoting pharmacists as antimicrobial stewardship proponents.

While we conducted this study utilizing a simulated patient method, which can be an effective method of deriving valid, reliable outcomes that are difficult to achieve by any other method in pharmacy practice research, our approach may have some limitations. First, the practice behavior in response to some simulated clinical scenarios may not be generalized to other clinical scenarios; this may preclude the evaluation of the pharmacists’ non-prescribed sale of antibiotics at pharmacies. As this interventional study recruited smaller amount of pharmacies from one urban area (Gondar town), our study may not be generalized to other practices of drug shops in rural areas, where alternative systems of medicine access may be more popular. The present study was conducted in community medicine retail outlets serving a relatively homogenous population. Future studies should consider recruiting community pharmacies from both rural and urban areas as the dispending behaviour of pharmacists could be affected by the geographical location of the pharmacy premises and the residents it serves. Regardless of the limitations mentioned, this study has significant implications in curbing the emergence of antimicrobial resistance via limiting the sale of antibiotics without a valid prescription in CMROs.

## Conclusions

The finding of the present study provides a basis to develop, execute and evaluate various comprehensive educational interventions aimed at providing correct, customized and evidence based information to pharmacists about antibiotic use and management of minor ailments. There is an urgent need of a paradigm shift among community pharmacists in Ethiopia to shift from commercial and business-based interests/practices to the provision of primary healthcare services to the community. Moreover, a stringent law/ policy enforcement regarding the sale of antibiotics without a valid prescription should be in place so as to make sure that pharmacy practices are in line with national guidelines for good dispensing practice.

## Additional file

Additional file 1: Non-prescribed sale of antibiotics for acute childhood diarrhea and upper respiratory tract infection in community pharmacies: a 2 phase mixed-methods study. (DOCX 23 kb)

## Abbreviations

AMR: Antimicrobial resistance
FMHACA: Food, Medicine and Healthcare Administration and Control Authority of Ethiopia
OR: Odds ratio
SPSS: Statistical Package for the Social Sciences
WHO: World Health Organization
