# Correction to: Non-prescribed sale of antibiotics for acute childhood diarrhea and upper respiratory tract infection in community pharmacies: a 2 phase mixed-methods study

**DOI:** 10.1186/s13756-018-0458-2

**Published:** 2019-01-11

**Authors:** Daniel Asfaw Erku, Sisay Yifru Aberra

**Affiliations:** 1 School of Pharmacy, University of Gondar, Lideta kebele 16, P.O.Box: 196, Gondar, Ethiopia; 2 College of Medicine and Health Sciences, University of Gondar, Gondar, Ethiopia

**Correction to: Antimicrob Resist Infect Control**

**https://doi.org/10.1186/s13756-018-0389-y**

The original article [1] contains a minor omission. The authors would like to note that the article cited in reference [2] in this Correction article should have been cited at the end of the following sentence in the Methods section of the original article:

“TPB has been employed extensively in pharmacy practice research.”

## References

[1] Erku DA, Aberra SY (2018). Non-prescribed sale of antibiotics for acute childhood diarrhea and upper respiratory tract infection in community pharmacies: a 2 phase mixed-methods study. Antimicrob Resist Infect Control.

[2] Amin M, Amine A, Newegy MS (2017). Perspectives of pharmacy staff on dispensing subtherapeutic doses of antibiotics: a theory informed qualitative study. Int J Clin Pharm.

